# Adjuvant Therapy of Oral Chinese Herbal Medicine for Menopausal Depression: A Systematic Review and Meta-Analysis

**DOI:** 10.1155/2018/7420394

**Published:** 2018-06-11

**Authors:** Jiju Wang, Jian Liu, Xiaojia Ni, Guangning Nie, Yuyan Zeng, Xiaojing Cao, Xiaoyu Li, Xiaoyun Wang

**Affiliations:** ^1^The Second Clinical School of Guangzhou University of Chinese Medicine, Guangzhou 510405, China; ^2^Guangdong Provincial Hospital of Chinese Medicine, The Second Affiliated Hospital to Guangzhou University of Chinese Medicine, Guangzhou 510120, China; ^3^School of Health and Biomedical Sciences, RMIT University, Bundoora, VIC 3083, Australia

## Abstract

*Objective. *The aim of this meta-analysis was to evaluate the effectiveness of oral Chinese herbal medicine (OCHM) combined with pharmacotherapy for menopausal depression.* Methods. *The electronic databases were searched from their inception to December 25, 2016, comprising PubMed, Embase, Cochrane Central Register of Controlled Trials, Chinese National Knowledge Infrastructure (CNKI), Wanfang database, and Chinese Biomedical (CBM) database. Randomized controlled trials investigating the effectiveness of OCHM combined with pharmacotherapy for the people with menopausal depression were eligible. Risk of bias was evaluated according to the Cochrane handbook. Meta-analyses were performed to pool the effect size. Heterogeneity and publication bias were also examined.* Results*. Twenty-two RCTs with 1770 participants were included in the review. None of the studies used placebo as the control and the risk of bias was high in blinding the participants and personnel. Overall, the meta-analysis demonstrated that adjuvant therapy of OCHM was effective in reducing the Hamilton Rating Scale for Depression (HAMD) scores compared to pharmacotherapy (MD = −3.75; 95% CI = −5.22, −2.29;* P* < 0.00001). The meta-analysis also suggested that OCHM adjuvant therapy for menopausal depression was superior to pharmacotherapy in terms of response rate of reducing HAMD scores (RR = 1.17; 95% CI = 1.10, 1.25; I2 = 55%).* Conclusions*. OCHM may provide additional effectiveness to pharmacotherapy for the people with menopausal depression. RCTs including the placebo control were required to further determine the additional efficacy of OCHM for menopausal depression.

## 1. Introduction

Depression is the most common mental disorder in menopausal women, with a prevalence ranging from 26% to 41.8% [1–5]. Menopause represents a significant transition in the life of a woman, and it has been considered as a specific window of vulnerability to depression [6, 7]. Women with menopausal depression are related to decreased quality of life, increased cardiovascular disease, and metabolic syndrome [8–11].

Antidepressants are considered the most common pharmacotherapy specifically for menopausal depression. However, the effect of antidepressants is unsatisfactory clinically, and the long-term use leads to considerable adverse effects. For example, selective serotonin reuptake inhibitors (SSRIs) are associated with sexual dysfunction, weight gain, and sleep disturbance [12–14]. Hormone therapy (HT) is widely used to alleviate menopause-related symptoms, and it has also been used in the treatment of menopausal depression [15, 16]. However, its effectiveness was inclusive [17]. Therefore, alternative therapies with more benefits and fewer harms were in continuous demand.

Traditional Chinese medicine (TCM) is one of the oldest medicine systems in the world and has been widely used as a form of complementary and alternative medicine [18, 19]. In China, oral Chinese herbal medicine (OCHM) is commonly used in combination with pharmacotherapy for menopausal depression and the associated clinical studies have been conducted [20, 21]. However, most of the clinical studies were of insufficient sample size and of varied quality of methodological design. Systematic reviews on OCHM for depression [22, 23] and menopausal-related symptoms have already been published [24]. However, previous systematic reviews did not specifically evaluate the effectiveness of OCHM as an adjunctive therapy to pharmacotherapy for menopausal depression.

This systematic review was motivated by the large number of published clinical trials on OCHM combined with pharmacotherapy and the unresolved problems of pharmacotherapy. Our objective was to evaluate the effectiveness of OCHM combined with pharmacotherapy for menopausal depression.

## 2. Materials and Methods

### 2.1. Search Strategy

We conducted and reported the systematic review according to the Preferred Reporting Items for Systematic Reviews and Meta-Analyses (PRISMA) statement guidelines [25]. The electronic databases were searched from their inception to December 25, 2016, comprising PubMed, EMBASE, Cochrane Central Register of Controlled Trials, Chinese National Knowledge Infrastructure (CNKI), Wan Fang Database, Chinese Scientific Journal Database (VIP), and Chinese Biomedical (CBM) database. The combination of search terms was presence of menopause, depression, traditional Chinese medicine, and randomized controlled trial (RCT). There was no limit to the language or publication status. The search strategy is attached in the Appendix.

### 2.2. Eligibility Criteria

1. *Types of Studies. *Only randomized controlled trials were eligible.
2. *Types of Participants. *Menopausal women aged 40 to 60 years with depressive symptoms were eligible. Depression was diagnosed according to the Classification and Diagnosis of Mental Diseases (CCMD-3) (Psychiatry Branch of Chinese Medical Association) [26], the Diagnostic and Statistical Manual (DSM-IV) (American Psychiatric Association) [27, 28], and the International Classification of Disease (ICD-10, WHO) (World Health Organization) [29].
3. *Types of Intervention and Control. *Studies that compared OCHM plus pharmacotherapy to the same pharmacotherapy were included. All forms of OCHM (i.e., decoctions, tables, capsules, pills, and powders) were included. The pharmacotherapy included conventional antidepressants (paroxetine hydrochloride, fluoxetine hydrochloride, deanxit, and venlafaxine), HT (estradiol valerate tablets and tibolone), and a combination of antidepressants and HT. The treatment duration had to last for three weeks at least.
4. *Types of Outcome Measurements. *The primary outcome was the total scores of Hamilton Rating Scale for Depression (HAMD). Clinically, HAMD scores have been commonly used to assess the severity of depression [30]. The secondary outcome was the response rate by reducing the scores of HAMD, Kupperman Menopausal Index (KMI), and adverse events (AEs).

### 2.3. Data Extraction and Quality Assessment

Titles and abstracts of all retrieved studies were screened, and then full texts were reviewed for eligibility by two authors (Jiju Wang and Jian Liu) independently. The data was collected by using the predefined data extraction form, including the journal title, first author, year of publication, size of study, baseline characteristics of women (e.g., age, course of disease), methodological design, intervention strategy, treatment duration, and outcomes. The dataset was validated by the third reviewer (Xiaouyn Wang).

Two reviewers independently evaluated the risk of bias by using the Cochrane Collaboration's tool [31]. Each domain of the bias was classified as a “low risk”, “unclear”, or “high risk”.

### 2.4. Data Synthesis

Meta-analysis was performed using Review Manager (the Cochrane Collaboration) software, Version 5.3.0. For dichotomous data, relative risk ratio (RR) with 95 % confidence intervals (CIs) was used to present the therapeutic effect; for continuous data, mean difference (MD) with 95 % CIs was used. The chi-square test and the Higgins I^2^ test were used to assess heterogeneity. If heterogeneity was low (I2 < 50% or P > 0.1), the fixed effects model was used. If heterogeneity was high (*I*^2^ > 50% or *P* < 0.1), the random effect model was used and further analyses such as subgroup or sensitivity analysis were planned to clarify the source of heterogeneity.

Publication bias was visualized by the funnel chart. Begg's correlation test and Egger's intercept test were used to quantify the publication bias, with a significant level at 0.05.

## 3. Results

### 3.1. Study Selection

**Figure 1:**
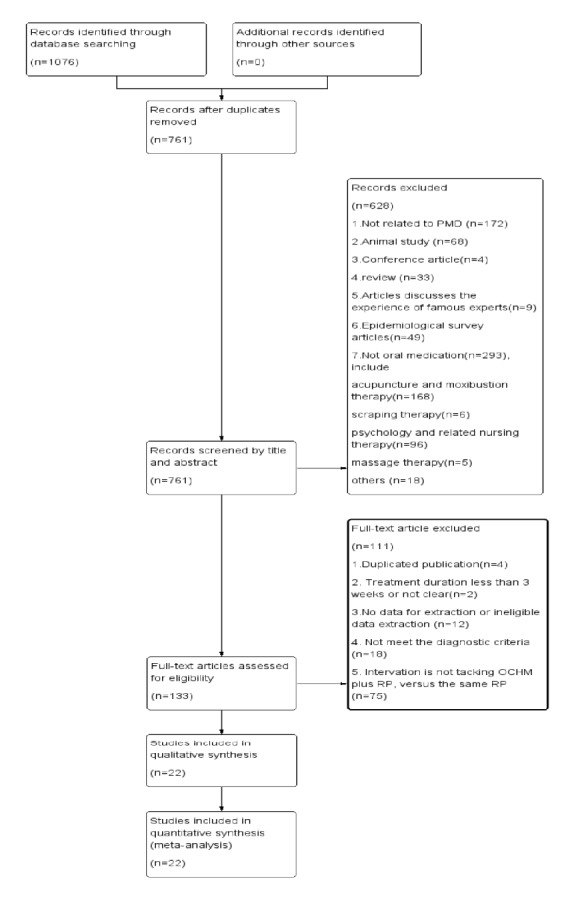
Flowchart of study selection.

Databases search found 1076 articles. After duplicates were removed, 761 articles were screened by reading the title/abstract and 628 ineligible articles were removed. After reading the full texts of the remaining 133 articles, 22 trials [32–53] were finally included in the systematic review. The flowchart summarizes the screening process (Figure 1).

### 3.2. Study Characteristics

All included trials were conducted in China and they were published ranging from 2005 to 2016. The total sample size was 1777, including 901 women in the experimental group and 876 women in the control group. The participants ranged between 40 and 60 years. The most common diagnostic instrument was CCMD-3, and the combination of CCMD-3 and HAMD score was usually used as the inclusion criterion of RCTs. For outcome measurements, eighteen trials reported HAMD score [32–34, 36–40, 42–46, 49–53], eighteen calculated response rate by reducing HAMD scores [33, 35–39, 41–49, 51–53], and three trials reported KMI score [39, 42, 45]. AEs were reported in 10 trails [32, 37–39, 41, 43, 45, 46, 51]. The basic characteristics of the included studies are summarized in Table 1.

Four forms of OCHM were investigated, consisting of decoctions, capsule, granule, and powder. Twenty-two formulas of OCHM were found. Bupleuri Radix (Chinese name: Chaihu) and Paeoniae Radix Alba (Chinese name: Baishao) were the herbs with top frequency. Details of OCHM in the included studies are summarized in Table 2. Control group included three categories of pharmacologic agents, antidepressants in 13 trials [32, 37–41, 44–49, 52], HT in 2 trials [42, 53], and the combination of antidepressants and HT in 6 trials [33–36, 50, 51]. One trial did not specify the pharmacotherapy [43]. The treatment duration ranged from three to 12 weeks.

### 3.3. Risk of Bias

**Figure 2:**
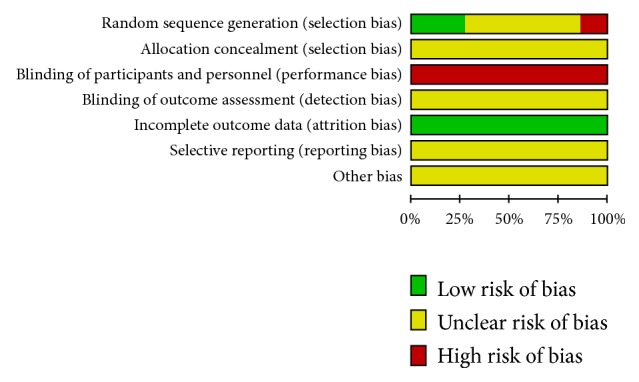
Risk of bias across included studies.

**Figure 3:**
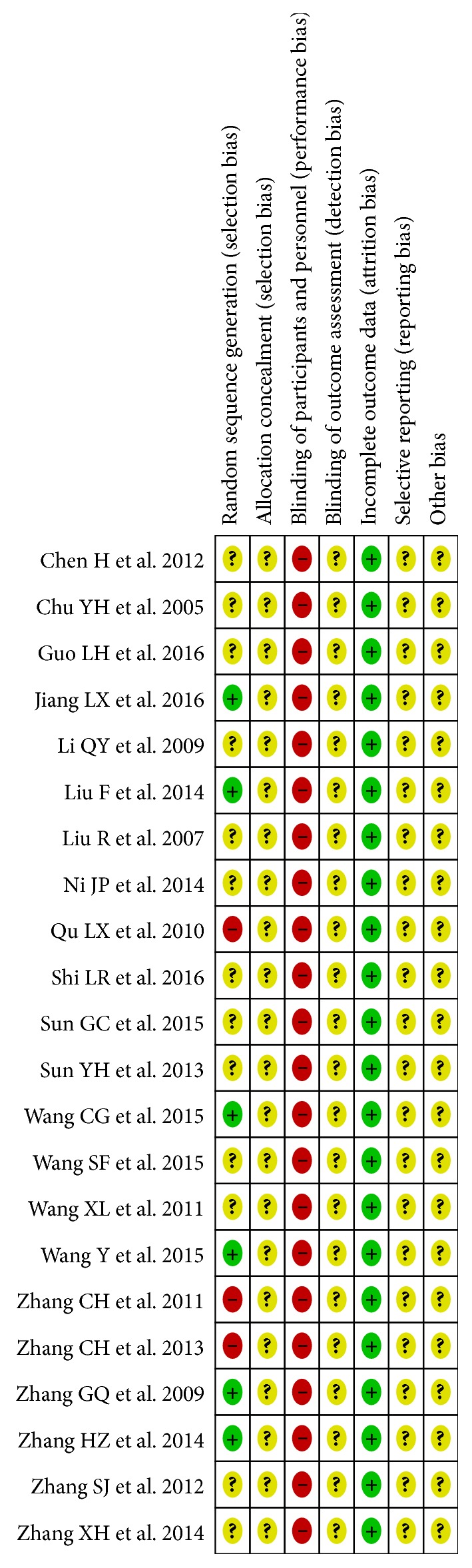
Risk of bias of individual studies. +: low risk of bias; ?: unclear risk of bias; −: high risk of bias.

The risk of bias was summarized in Figures 2 and 3. For random sequence generation, six trials used random number tables [32, 39, 42, 45, 46, 52], and 13 trials [33–35, 37, 38, 40, 41, 43, 47–50, 53] did not provide details of randomization. Three trials considered the order of visits as random, which were of high risks in selection bias [36, 44, 51]. None of the trials reported allocation concealment. Binding of participants, personnel, and outcome assessors was not applied in any studies. None of the studies had attribution bias. The risk of bias in selective reporting was unclear as none of the studies published their protocols.

### 3.4. Effect of Intervention

#### 3.4.1. Hamilton Rating Scale for Depression (HAMD) Scores

**Figure 4:**
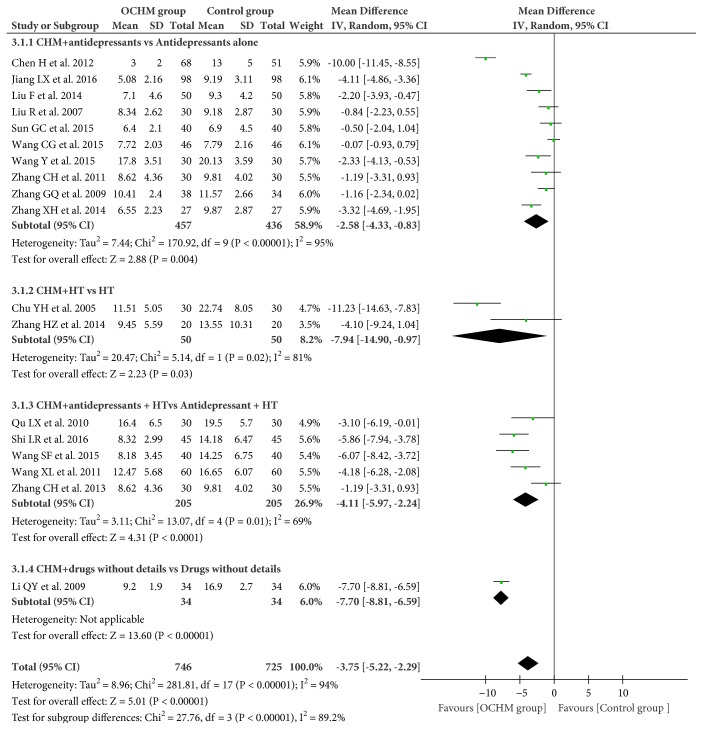
Forest plot of HAMD scores. HAMD: Hamilton Rating Scale for Depression; CHM: Chinese herbal medicine; HT: hormone therapy.

Eighteen RCTs (n = 1417 participants) used HAMD scores to measure the effect of OCHM for menopausal depression [32–34, 36–40, 42–46, 49–53]. Overall, the adjuvant use of OCHM therapy in pharmacotherapy was superior to pharmacotherapy alone (mean difference (MD) = −3.75; 95% CI = −5.22, −2.29; I^2^ = 94%; random model) (Figure 4).

As the meta-analysis was heterogeneous, subgroup analysis in terms of different categories of pharmacologic agents was performed (Figure 4). The subgroup analysis showed that adjuvant therapy of OCHM for menopausal depression was more effective than antidepressants alone (MD = −2.58; 95% CI = −4.33, −0.83; I^2^ = 95%) [32, 37–40, 44–46, 49, 52]. It was superior to HT alone (MD = −7.94; 95% CI = −14.90, −0.97; I^2^ = 81%) [42, 53] and antidepressants combined with HT (MD = −4.11; 95% CI = −5.97, −2.24; I^2^ = 69%; random model) [33, 34, 36, 50, 51]. Another study [43] did not specify the pharmacotherapy.

#### 3.4.2. Response Rate

**Figure 5:**
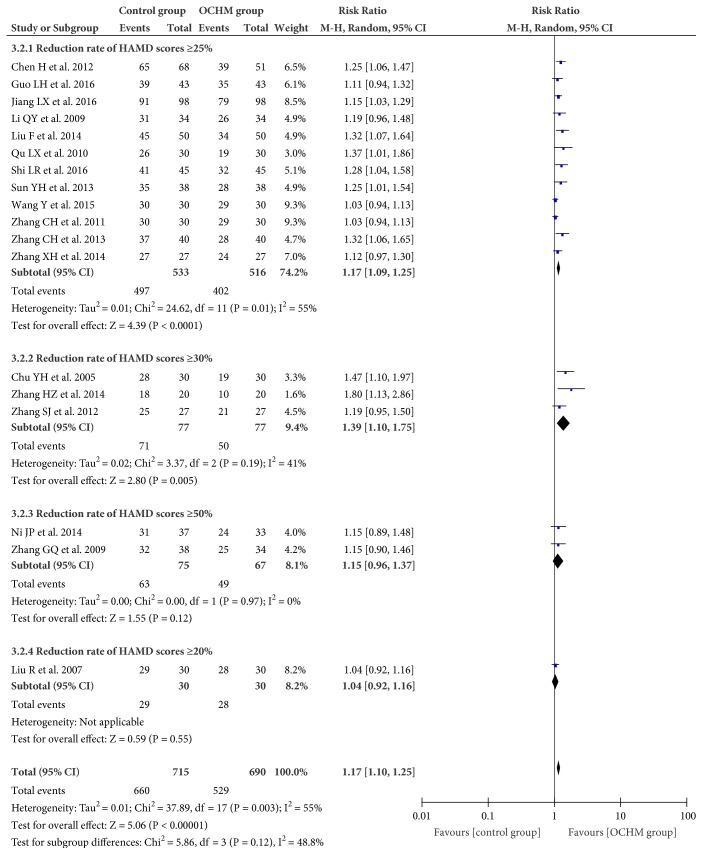
Forest plot of response rate.

Eighteen trials (n = 1189 participant) applied response rate of reducing HAMD scores in outcome measurement [33, 35–39, 41–49, 51–53]. The overall meta-analysis showed OCHM adjuvant therapy for menopausal depression was superior to pharmacotherapy (RR = 1.17; 95% CI = 1.10, 1.25; I^2^ = 55%; random model) (Figure 5).

#### 3.4.3. Kupperman Menopausal Index (KMI) Scores

**Figure 6:**
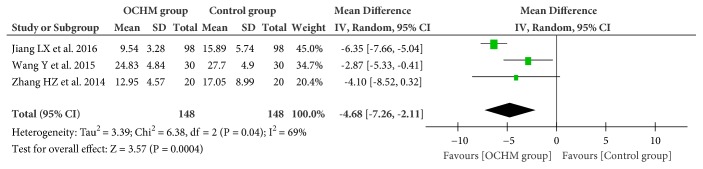
Forest plot of Kupperman menopausal index scores.

Three RCTs [39, 42, 45] measured the effectiveness of OCHM for menopausal depression by using KMI scores. Meta-analysis showed OCHM combined with pharmacotherapy was more effective than pharmacotherapy alone (MD = −4.68; 95% CI = −7.26, −2.11; I^2^ = 69%; random model) (Figure 6).

#### 3.4.4. Adverse Events (AEs)

**Figure 7:**
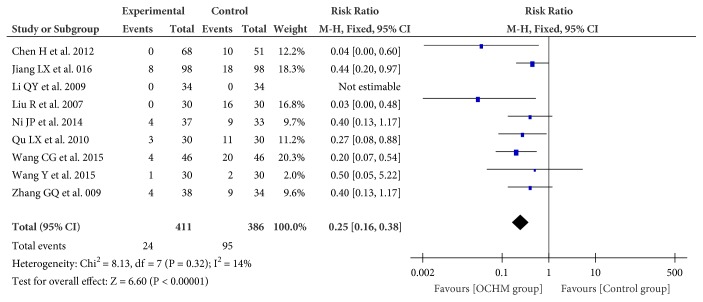
Forest plot of adverse event.

AEs were monitored in nine studies (n = 797 participants) [32, 37–39, 41, 43, 45, 46, 51]. Meta-analysis showed that the incident of adverse events in the group of OCHM plus pharmacotherapy was less than the pharmacotherapy (RR = 0.25; 95% CI = 0.16, 0.38; I^2^ = 14%; fixed model) (Figure 7).

### 3.5. Publication Bias

**Figure 8:**
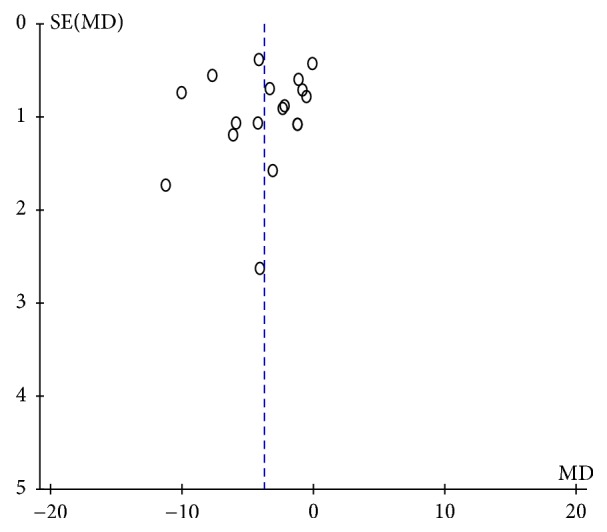
Funnel plot of HAMD scores.

**Figure 9:**
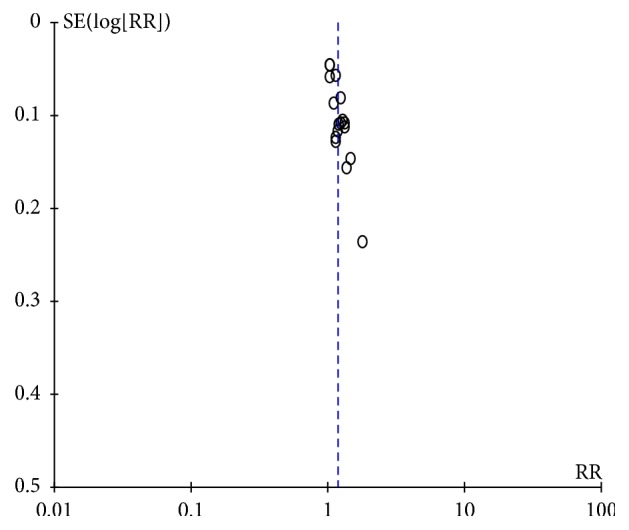
Funnel plot of response rate.

The funnel plots of HAMD scores and the response rate were asymmetrical (Figures 8 and 9). The regression analysis of Begg's rank correlation test and Egger's intercept test was statistically significant (*P* > 0.05), suggesting the presence of publication bias. The detection of publication bias was not available for other outcomes as the included studies were less than 10.

## 4. Discussion

### 4.1. Statement of Principal Findings

In the present study, we reviewed 22 RCTs involving a total of 1777 participants and assessed the add-on effects and safety of OCHM to pharmacotherapy in women with menopausal depression. Meta-analysis showed that the combination of OCHM and pharmacotherapy was more effective in improving menopausal depression. In addition, the incidence of AEs in the participants treated with OCHM adjunctive therapy was less than those without OCHM adjunctive therapy. However, the risk of bias of the included RCTs may affect the evidence certainty; particularly most studies did not blind the participants or personnel.

### 4.2. Possible Explanations for the Evidence

OCHM can increase the expression level of estrogen receptors in hypothalamic pituitary ovarian (HPO) axis [54, 55]. OCHM can also alleviate hippocampal neuron damage, inhibit early apoptosis of neurons, and increase the content of monoamine transmitters in brain tissue through the CREB-BDNF signaling pathway [56]. Furthermore, OCHM can regulate the hypothalamus pituitary adrenal (HPA) axis in menopausal depression rats and improve the behavior of model rats [57]. Bupleuri Radix (Chinese name: Chaihu) and Paeoniae Radix Alba (Chinese name: Baishao) were the herbs with top frequency. Bupleurum-saikoside, the main active ingredient of Bupleuri Radix, improved depression by regulating the monoamine neurotransmitters and BDNF in the brain [58]. Paeoniae Radix Alba improved depression by increasing the single amine neurotransmitter and adjusting the dysfunction of HPA axis [59].

### 4.3. Limitations and Implications of the Research

There were several limitations in this study. Firstly, the quality of the included trials was generally poor. The risks of selection bias and reporting bias were unclear most of the time. And the risk of performance bias in almost all the RCTs was high. These biases may affect the results of meta-analysis. Secondly, the heterogeneity was observed across meta-analyses and it was not resolved by subgroup analysis. This can also reduce the evidence certainty. Thirdly, none of the RCTs included considered placebo as the control. Hence the current studies were unable to conclude the efficacy of OCHM. Although it is difficult to successfully produce a placebo to CHM because of its special characteristics such as the appearance, smell, and taste, recent progress of placebo making can be applied. For example, when researchers explored the preparation method of placebo to Moron Dan, they found that the flavor characteristics and disintegration of the placebo made of soybean powder 100g, starch 100g, carbon black pigment 1g, and honey 70g were similar to the experimental drug [60]. Some researchers suggested that encrypted capsules could be also used as a placebo [61].

## 5. Conclusion

Adjuvant therapy of OCHM provided additional benefits to pharmacotherapy in the people with menopausal depression. More RCTs with a rigorous design, particularly applying placebo as the control as well as blinding the participants and personnel, are needed to confirm the efficacy of OCHM for menopausal depression.

## Figures and Tables

**Table 1:** Basic characteristics of the included studies.

Reference	Sample size(I/C)	Age(I/C)	Duration of disease(I/C)	Diagnosis standard	Intervention group	Control group	Treatment duration	*Outcome*
Chen H, 2012	68/51	42-59 (50.20 ± 3.94) /43-58 (50.78 ± 4.08)	1.76 ± 0.99 y /1.91 ± 1.25 y	Age; CCMD-3; HAMD	OCHM + antidepressant	antidepressant	8w	①②④

Chu YH, 2005	30/30	41-60 (47.86 ± 4.42) /41-60 (48.3 ± 4.06)	NS	Age; CCMD-3; HAMD	OCHM + HT	HT	6w	①②

Guo LH, 2016	43/43	40-55/42-55	(1.8 ± 0.6) y/(1.6 ± 0.8) y	Age; CCMD-3; HAMD	OCHM + antidepressant	antidepressant	6w	②

Jiang LX, 2016	98/98	41-60 (51.18 ± 4.52)/46-54(48.85 ± 3.27)	(4.8 ± 1.7) y/(4.2 ± 1.0) y	Age; CCMD-3; HAMD	OCHM + antidepressant	antidepressant	8w	①②③④

Li QY, 2009	34/34	41-56 (46.8 ± 4.1)	NS	Age; ICD-10; HAMD	OCHM + pharmacotherapy	pharmacotherapy	4w	①②④

Liu F, 2014	50/50	44-56 (49.3 ± 2.4) /43-57 (48.4 ± 2.3)	(2.3 ± 0.5) y/(2.1 ± 0.4)y	Age; ICD-10	OCHM + antidepressant	antidepressant	8w	①②

Liu R, 2007	30/30	(54.32 ± 3.29) /(54.0 ± 4.62)	(11.32 ± 6.25) m/(12.12 ± 4.58)m	Age; CCMD-3; HAMD	OCHM + antidepressant	antidepressant	8w	①②④

Ni JP, 2014	37/33	(52 ± 4)/(52 ± 4)	(9 ± 5) m/ (9 ± 5) m	Age; CCMD-3; HAMD	OCHM + antidepressant	antidepressant	4w	②④

Qu LX, 2010	30/30	45-55(51.6 ± 3.2) /45-55(50.8 ± 2.8)	(10.5±2.7)m /(11.1±5.2)m	Age; CCMD-3; HAMD	OCHM + HT + antidepressant	HT + antidepressant	8w	①②④

Shi LR, 2016	45/45	41-58(46.4 ± 4.2) /42-57(44.8 ± 3.3)	(11.7 ± 4.5)m /(10.6 ± 6.3)m	Age; CCMD-3; HAMD	OCHM + HT + antidepressant	HT + antidepressant	12w	①②

Sun GC, 2015	40/40	42.2 ± 2.60 /42.2 ± 2.60	(1.92 ± 1.06) y /NS	Age; ICD-10; HAMD	OCHM + antidepressant	antidepressant	4w	①

Sun YH, 2013	38/38	43-48(44.76 ± 2.24) /42-49(45.02 ± 3.15)	(2.38 ± 1.24) y /(2.54 ± 1.56)y	Age; CCMD-3; HAMD	OCHM + HT + antidepressant	HT + antidepressant	4w	②

Wang CG, 2015	46/46	44-53(46.93 ± 6.8) /44-53(47.52 ± 7.2)	NS	Age; CCMD-3	OCHM + antidepressant	antidepressant	6w	①④

Wang SF, 2015	40/40	43-50(46.45 ± 6.15) /42-49(45.82 ± 5.45)	(2.75 ± 0.35)y /(2.85 ± 0.43)y	Age; CCMD-3; HAMD	OCHM + HT + antidepressant	HT + antidepressant	3w	①

Wang XL, 2011	60/60	45-55(51.21 ± 3.17) /45-55(50.86 ± 3.41)	NS	Age; CCMD-3; HAMD	OCHM + HT + antidepressant	HT + antidepressant	8w	①

Wang Y, 2015	30/30	45.20 ± 2.90 /44.80 ± 3.10	NS	Age; ICD-10; HAMD	OCHM + antidepressant	antidepressant	8w	①②③④

Zhang CH, 2013	40/40	43-56(46.80 ± 4.10) /44-55(45.40 ± 3.2)	(11.50±4.30)m /(10.90±6.50)m	Age; CCMD-3; HAMD	OCHM + HT + antidepressant	HT + antidepressant	3w	①②

Zhang CH, 2011	30/30	45-55(43.50 ± 12.43) /45-55(42.25 ±11.38)	NS	Age; CCMD-3; HAMD	OCHM + antidepressant	antidepressant	6w	①②

Zhang GQ, 2009	38/34	45-56(51.63 ± 3.68) /45-56(51.95 ± 3.86)	(9.44 ± 4.69)m /(8.88 ± 4.65) m	Age; CCMD-3; HAMD	OCHM + antidepressant	antidepressant	4w	①②④

Zhang HZ, 2014	20/20	45-53 /46-52	(6-24)m /(5-22)m	Age; CCMD-3	OCHM + HT	HT	12w	①②③

Zhang SJ, 2012	27/27	43-54(46.5 ± 6.3) /44-55(45.3 ± 7.2)	(3.2 ± 2.8)m /(4.1 ± 3.2)m	Age; DSM-IV	OCHM + antidepressant	antidepressant	4w	②

Zhang XH, 2014	27/27	(47 ± 6.8) /(46.0 ± 6.8)	NS	Age; CCMD-3; HAMD	OCHM + antidepressant	antidepressant	8w	①②

CCMD-3: Criteria for Classification and Diagnosis of Mental Diseases; DSM-IV: Diagnostic and Statistical Manual; ICD-10: International Classification of Disease; m = month; y = year; NS: not stated.OCHM: oral Chinese herbal medicine; HT: hormone therapy; w = week; ①: Hamilton Rating Scale for Depression (HAMD) score; ②: response rate; ③: Kupperman Menopausal Index (KMI) score; ④: adverse events (AEs).

**Table 2:** Chinese herbal medicine of the included studies.

Study	Formula	^a^ Herbal ingredients	Preparation	Dosage	Frequency
Chen H, 2012	Jie Yu Jing Xin Ke Li	zhenzhumu, huaixiaomai, shoudihuang, shanzhuyu, tusizi, suanzaoren, fuling, chaihu, baishao, meiguihua, danggui, nvzhenzi	granule	1 bag	bid

Chu YH, 2005	Xiao Yao Jie Yu Tang	Chaihu, danggui, baishao, baizhu, fuling, weijiang, bohe, zhi gan cao, xian ling pi, nvzhenzi, shengmaiya, chaomaiya	decoction	1 pack decocted twice	bid

Guo LH, 2016	Bai He Di Huang Tang	Baihe, sheng di huang, long gu, muli, danggui, he huan pi, chaihu, ye jiao teng, fushen, yujin, zhi mu	decoction	1 pack decocted twice	bid

Jiang LX, 2016	Wu Ling Jiao Nang	wulingjun	capsule	3 tablets	tid

Li QY, 2009	Jie Yu Tang	Dangshen, chuanxiong, danggui, danshen, xiangfu, he huan pi, gualou, yujin	decoction	1 pack decocted twice	bid

Liu F, 2014	Suan Zao Ren Tang	Suanzaoren, chuanxiong, fuling, wuweizi, zhi mu, gan cao	decoction	1 pack decocted twice	bid

Liu R, 2007	Xue Fu Zhu Yu Jiao Nang	Taoren, honghua, chishao, chuanxiong, zhiqiao, chaihu, jiegeng, danggui, dihuang, niuxi, gan cao	capsule	6 tablets	bid

Ni JP, 2014	Shen Song Yang Xin Jiao Nang	Renshen, maidong, shanzhuyu, danshen, suanzaoren, sangjishen, chishao, tu bie chong, gansong, huanglian, wuweizi, long gu	capsule	4 tablets	tid

Qu LX, 2010	Jie Yu Zi Shen Tang	Chaihu, xiangfu, baishao, baizhu, yujin, gan cao, suanzaoren, fuling, shichangpu, shoudihuang, shanyurou, shanyao	decoction	1 pack decocted twice	bid

Shi LR, 2016	Zi Ni Zi Gan Yang Shen Tang	Fuling, gan cao, baishao, chaihu, chuanxiong	decoction	1 pack decocted twice	bid

Sun GC, 2015	Jia Wei Xiao Yao San	Chaihu, baizhu, bohe, danggui, fuling, baishao, gan cao, shengjiang, zhizi, danpi	granule	1 bag	tid

Sun YH, 2013	Zi Ni Shu Gan Jie Yu Tang	Chaihu, xiangfu, chuanxiong, chishao, yujin, chenpi, sheng di huang, shanzhuyu, danpi, yuanzhi, gan cao	decoction	1 pack decocted twice	bid

Wang CG, 2015	Zi Ni An Shen Jie Yu Tang	Huangqi, huangqin, chaihu, muxiang, zhiqiao, gan cao, sharen, peilan, dangshen, fushen, danggui, baizhu, chuanxiong, yujin, suanzaoren, ye jiao teng, long yan rou	decoction	1 pack decocted twice	bid

Wang SF, 2015	Zi Ni Shu Gan Jie Yu Tang	Chaihu, xiangfu, chuanxiong, baishao, yujin, chenpi, zhi mu, shengdi, shanzhuyu, danpi, suanzaoren, gan cao	decoction	1 pack decocted twice	bid

Wang XL, 2011	Si Hua Jie Yu Tang	He huan hua, xuanhua, meiguihua, baimeihua, suanzaoren, bai zi ren, fuxiaomai, fushen, ye jiao teng, tiandong, maidong, wuweizi, gan cao, dazao	decoction	1 pack decocted twice	bid

Wang Y, 2015	Zi Ni Bai He Di Huang Tang	Baihe, shengdi, maidong, wuweizi, he huan pi, ye jiao teng, fuling, yuanzhi, shichangpu, yujin, chuanxiong, gan cao	decoction	1 pack decocted twice	bid

Zhang CH, 2013	Wu Ling Jiao Nang	wulingjun	capsule	3 tablets	tid

Zhang CH, 2011	Zi Ni Zi Yin Bu Shen Tang	Chaihu, xiangfu, chuanxiong, baishao, yujin, shoudihuang, fuling, shanzhuyu, suanzaoren, gan cao	decoction	1 pack decocted twice	bid

Zhang GQ, 2009	Zi Ni Zao Ren Bu Xue Tang	Suanzaoren, fuling, chuanxiong, zhi mu, gan cao, huangqi, danggui, shanzhuyu, shoudihuang, danshen, chaihu, xiangfu, yujin, shichangpu	decoction	1 pack decocted twice	bid

Zhang HZ, 2014	Si Er Wu He Fang And Gan Mai Da Zao Tang	Danggui, baishao, danshen, shoudihuang, xianmao, xian ling pi, fu pen zi, tusizi, wuweizi, che qian zi, gouqi, yujin, huanglian, rougui, xiaomai, gan cao, dazao	decoction	1 pack decocted twice	bid

Zhang SJ, 2012	Zi Ni Bai He Di Huang Tang	Baihe, shengdi, danshen, chuanxiong, juhua	decoction	1 pack decocted twice	bid

Zhang XH, 2014	Kun Tai Jiao Nang	Shoudihuang, huanglian, baishao, huangqin, ejiao, fuling	capsule	4 tablets	tid

^a^ The herbal ingredients are presented as Chinese pinyin. Bid: twice per day; tid: three times per day.

## Appendix

Search strategy included the following:

- #1 Menopause [Mesh]
- #2 ((((((((((Perimenopausal [ti.ab]) OR menopausal [ti.ab]) OR menopause [ti.ab]) OR perimenopause [ti.ab]) OR premenopause [ti.ab]) OR postmenopause [ti.ab]) OR menopausal transition[ti.ab]) OR premenopausal [ti.ab]) OR postmenopausal [ti.ab]) OR climacterium [ti.ab]) OR climacteric [ti.ab]
- #3 #1 OR #2
- #4 Depression [Mesh]
- #5 (((((((((Depression [ti.ab]) OR Depressions [ti.ab]) OR Depressive Symptoms [ti.ab]) OR Depressive Symptom [ti.ab]) OR Symptom, Depressive [ti.ab]) OR Symptoms, Depressive [ti.ab]) OR Emotional Depression [ti.ab]) OR Depression, Emotional [ti.ab]) OR Depressions, Emotional [ti.ab]) OR Emotional Depressions [ti.ab]
- #6 #4 OR #5
- #7 Medicine, Chinese Traditional [Mesh]
- #8 ((((((Medicine, Chinese Traditional [ti.ab]) OR Traditional Chinese Medicine [ti.ab]) OR Chinese Medicine, Traditional [ti.ab]) OR Chung I Hsueh [ti.ab]) OR Hsueh, Chung I [ti.ab]) OR Zhong Yi Xue [ti.ab]) OR Chinese Traditional Medicine [ti.ab]) OR Traditional Medicine, Chinese [ti.ab]
- #9 #7 OR #8
- #10 ((randomized controlled trial [Publication Type]) OR randomized [ti.ab]) OR placebo [ti.ab]
- #11 #3 AND #6 AND #9 AND #10
